# Supplementary material for: A Multiomics Evaluation of the Countermeasure Influence of 4-Week Cranberry Beverage Supplementation on Exercise-Induced Changes in Innate Immunity
Source: Nutrients. 2024 Sep 26;16(19):3250. doi: 10.3390/nu16193250 (PMC11479082; doi:10.3390/nu16193250)
Supplement: Supplementary file 1 [file nutrients-16-03250-s001.zip › nutrients-3212546-supplementary table S1.pdf]

nutrients

MDPI

A Multiomics Evaluation of the Countermeasure Influence of 4-Week Cranberry Beverage Supplementations on Exercise-Induced Changes in Innate Immunity

David C. Nieman [1,*], Camila A. Sakaguchi [1], James C. Williams [1], Jongmin Woo [2], Ashraf M. Omar [2], Fayaj A. Mulani [2], Qibin Zhang [2], Wimal Pathmasiri [3,4], Blake R. Rushing [3,4], Susan McRitchie [3], Susan J. Sumner [3,4], Jackie Lawson [5] and Kevin C. Lambirth [5]

Supplementary Table S1. Polyphenol and flavonoid content of the cranberry and placebo beverages. Data are expressed as mean ± SE from four batch measurements except for selected flavonoids measured once via liquid chromatography quadrupole time-of-flight.

Polyphenols and Flavonoids	Cranberry Beverage (mg/240 ml)	Placebo (mg/240 ml)
*Cyanidin-3-Arabinoside	28.1±3.5	ND
*Cyanidin-3-Galactoside	49.8±2.4	ND
*Cyanidin-3-Glucoside	<6	ND
*Peonidin-3-Arabinoside	24.3 ± 2.4	ND
*Peonidin-3-Galactoside	67.8 ± 10.0	ND
*Peonidin-3-Glucoside	5.2 ± 1.1	ND
*Total Anthocyanins	41.4 ± 4.5	ND
**Proanthocyanidins (PACs)	294 ± 26.1	ND
***Phenolic acids	3.48	1.28
***Catechins	2.04	1.28
***Flavonols	33.24	0
****Total Polyphenols	316.5 ± 18.9	23.3 ± 5.2

** High-Performance Liquid Chromatography with Ultraviolet Detection (HPLC-UV)*
*** Colorimetric assay using dimethylaminocinnamaldehyde (DMAC)*
**** Liquid Chromatography Quadrupole Time-of-Flight (LC-QTOF)*
***** Folin–Ciocalteu (F–C) assay*
